# Increased amounts and stability of telomeric repeat-containing RNA (TERRA) following DNA damage induced by etoposide

**DOI:** 10.1371/journal.pone.0225302

**Published:** 2019-11-22

**Authors:** Bong-Kyeong Oh, Yoojung Choi, Jaeman Bae, Won Moo Lee, Jeong-Kyu Hoh, Joong Sub Choi

**Affiliations:** 1 Institute for the Integration of Medicine and Innovative Technology, Hanyang University College of Medicine, Seoul, Korea; 2 Department of Translational Medicine, Hanyang University Graduate School of Biomedical Science and Engineering, Seoul, Korea; 3 Department of Obstetrics and Gynecology, Hanyang University College of Medicine, Seoul, Korea; Tulane University Health Sciences Center, UNITED STATES

## Abstract

Telomeric repeat-containing RNAs (TERRAs) are long noncoding RNAs transcribed from subtelomeres toward telomeric repeat tracts, which have been implicated in telomere protection and heterochromatin formation. Genotoxic stress leads to upregulation of TERRAs. However, the mechanism of DNA damage-mediated TERRA induction remains elusive. Here, we treated HeLa cells with etoposide, a DNA double-strand break-generating agent, for various times and monitored the levels of TERRAs. Etoposide treatment led to a gradual time-dependent increase in TERRAs. Etoposide-mediated induction was evident in many TERRAs arising from various chromosome loci, including 20q and XpYp. Chromatin immunoprecipitation assays revealed no significant changes in the occupancy of RNA polymerase II at telomeres upon etoposide treatment. Interestingly, TERRAs arising from 20q, XpYp, 10q, and 13q degraded at slower rates in cells treated with etoposide, while degradation rates of TERRAs from many loci tested were nearly identical in both etoposide- and mock-treated cells. Telomere damage occurred from early time points of etoposide treatment, but telomere lengths and abundance of telomeric repeat-binding factor 2 (TRF2) at telomeres remained unchanged. In summary, etoposide treatment led to telomere damage and TERRA accumulation, but telomere lengths and TRF2-mediated telomere integrity were maintained. Etoposide-mediated TERRA accumulation could be attributed partly to RNA stabilization. These findings may provide insight into the post-transcriptional regulation of TERRAs in response to DNA damage.

## Introduction

Telomeres protect chromosome ends from DNA damage [1]. Human telomeres are composed of tandem 5′–TTAGGG–3′ repeats and are bound by shelterin complexes composed of six proteins [2]. Telomeric repeat-binding factor 2 (TRF2), a component of shelterin, binds directly to telomeric DNA repeats and plays essential roles in telomere protection. Abrogation of TRF2 results in destruction of telomere structure, a high incidence of end-to-end chromosome fusions, and cell death [3–5]. Telomeric erosion occurs during cell division, presumably because of the “DNA end-replication problem” [6,7]. Short telomeres trigger DNA damage signals that lead to growth arrest and replicative senescence [8].

Telomeres are transcribed into long noncoding RNAs termed telomeric repeat-containing RNAs or TERRAs [9,10]. Transcription of TERRAs is mediated by RNA polymerase II (RNAPII), which is initiated within subtelomeres toward the telomeric tract. TERRAs comprise subtelomere-specific sequences and UUAGGG repeats, and their sizes are heterogeneous from a few hundreds to >10 kb [9,10]. A significant proportion of TERRAs remains bound with telomeres, suggesting that they are an integral part of telomere heterochromatin [9–12]. Indeed, TERRAs have been implicated in telomere protection [13–16], heterochromatin formation [16], and telomere replication [17,18]. Importantly, decrease in the levels of TERRAs led to loss of cellular viability in cancer cells, suggesting that TERRAs play essential roles in cell viability [13]. TERRA has been reported to be expressed from several chromosome ends [19,20]. Recently, Feretzaki *et al*. demonstrated that TERRA is expressed from numerous chromosome ends and that the relative expression values vary among the different cell types [21]. In contrast, Montero *et al*. have reported that transcripts arising from 20q and XpYp loci have genuine TERRA features and that the chromosome 20q locus is a major TERRA source in human [13].

Upregulation of TERRAs occurs following cellular stresses such as heat shock and genotoxic stress [10,19,22]. Indeed, heat shock factor 1, a transcription factor, is known to be involved in upregulation of TERRAs by binding to subtelomeric regions upon heat shock [22]. TERRAs accumulate upon treatment with zeocin, an agent generating DNA double-strand breaks (DSBs), which was suggested to be caused by the loss of TRF2 at telomeres [19]. TRF2 was found to repress transcription of TERRAs through its homodimerization domain [19], which is important for chromatin compaction [23,24]. However, the mechanism of genotoxic stress-mediated regulation of TERRAs is largely unknown.

Here, we treated HeLa cells with etoposide—another type of DSB-inducing agent—for various times and monitored the levels of TERRAs. We found time-dependent increases in TERRAs, which were detected at many chromosome loci but not all. We observed occurrence of telomere damage upon etoposide treatment, but no significant differences in telomere lengths or TRF2 abundance at telomeres. We also examined the stability of TERRAs and the telomere-binding activity of RNAPII, which revealed that RNA stabilization accounted partly for the accumulation of TERRAs.

## Materials and methods

### Cell culture

HeLa cells purchased from Korean Cell Line Bank (KCLB; Seoul, Korea) were maintained in Dulbecco’s modified Eagle’s medium (Welgene, Daegu, Korea) supplemented with 10% heat inactivated fetal bovine serum (GIBCO, Grand Island, NY, USA) and 1% streptomycin and penicillin (GIBCO) at 37 °C and 5% CO_2_ in humidified air. For the treatment of actinomycin D, HeLa cells were treated with dimethyl sulfoxide (DMSO, vehicle alone; Sigma-Aldrich, St. Louis, MO, USA) or 100 μM of etoposide (Tokyo Chemical Industry, Tokyo, Japan); 24 h later the medium was replaced with fresh, and actinomycin D was added at 5 μg/mL for the indicated times.

### MTT cell viability assays

Cells were seeded in 96-well plates in triplicate at a density of 5,000 per well, incubated overnight, and supplemented with 100 μM of etoposide for the indicated times. MTT (5 mg/mL in phosphate-buffered saline; Duchefa, Haarlem, The Netherlands) was added to each well at a concentration of 100 μg and incubated at 37 °C for 3–3.5 h under 5% CO_2_ in humidified air. The reaction was stopped by the addition of 100 μL DMSO after the medium had been removed. Cells were agitated on an orbital shaker for 15 min in the dark, and cell viability was determined by measuring absorbance at 550 nm using a SpectraMax microplate reader (Molecular Devices, Sunnyvale, CA, USA).

### Preparation of digoxigenin (DIG)-labeled probes

Terminal transferase was used to label oligonucleotides at their 3′- ends by incorporation of a single DIG-labeled ddUTP (Roche Diagnostics, Mannheim, Germany). Briefly, 200 pmol oligonucleotides were incubated with 1 μL of 1 mM DIG-ddUTP and 20 U of terminal transferase (Roche) in 1 × reaction buffer and 5 mM CoCl_2_ to a final volume of 20 μL at 37 °C for 60 min, and the reaction was stopped by adding 2 μL 0.2 M EDTA, pH 8.0. For the preparation of 18S rRNA probes, 18S rDNA was labeled by random priming with DIG-dUTP using DIG-High Prime kits (Roche). One microgram of 18S rDNA, amplified by polymerase chain reaction (PCR) and purified by agarose gel extraction, was mixed with the 5 × random primer mix supplied, and incubated at 37 °C overnight. The primers used for 18S rDNA amplification by PCR were 5′–AAC CTC GGG CCG ATC GCA CG–3′ and 5′–TCA AAG TAA ACG CTT CGG GC–3′.

### Southern blot analysis of terminal restriction fragment lengths

Genomic DNA was isolated using proteinase K treatment and phenol/chloroform/isoamyl alcohol extraction [25]. Telomere length was measured using Southern hybridization, as described previously [26]. Briefly, 5 μg of HinFI-cut DNA was fractionated by agarose gel electrophoresis and blotted onto nylon membranes (Hybond^™^ N+; GE Healthcare Biosciences, Buckinghamshire, UK) in 10 × saline–sodium citrate (SSC) buffer using capillary transfer. After ultraviolet (UV) crosslinking (Spectronics, Westbury, NY, USA), membranes were hybridized with DIG-labeled d(TTAGGG)4 in DIG Easy Hyb^™^ (Roche) at 45 °C overnight. The membranes were washed and DIG detected as described in the Roche user’s guide (catalog No. 11093657910). The hybridization signal was detected with CDP-*star* (Roche) by scanning using a ChemiDoc XRS+ image analysis system (Bio-Rad, Hercules, CA, USA). The membrane was rinsed thoroughly with sterile water, washed at 37 °C in stripping buffer (0.2 N NaOH; 0.1% SDS) for 2 × 15 min, and then rinsed with 2 × SSC buffer for 5 min. The deprobed blot was rehybridized with the DIG-labeled d(CACCAC)_4_ probe. The mean telomere length (MTL) was calculated as described [27]. Briefly, the telomere signal in each lane was quantified in a grid object defined as a single column with 25 rows using Image Lab^™^ software (Bio-Rad, Version 6) (1 × 25 grid over the lane) and the MTL was defined as ∑(MWi × ODi)/∑(ODi), where ODi is the densitometer output and MWi is the length of the DNA at position ‘i’.

### Reverse transcription quantitative polymerase chain reaction (RT–qPCR)

Total RNA, 10–20 μg aliquots, isolated using TRI-RNA (Favorgen, Taipei, Taiwan) or TRIzol^®^ reagent (Thermo Fisher Scientific, Waltham, MA, USA), were incubated with 5 U RNase-free DNase I (New England Biolabs, NEB, Ipswich, MA, USA) and 60 U RNase inhibitor (Takara, Kyoto, Japan) in 2 mM DTT at 37 °C for 30 min, and the DNase I reaction was repeated once. RNA integrity was evaluated by agarose gel electrophoresis. cDNA synthesis for TERRA was performed as described with slight modifications [28]. Briefly, 3 μg of DNase I-treated RNA was incubated with 2 U RNase-free DNase I (NEB) and 40 U RNase inhibitor (Takara) in 2 mM DTT in a total volume of 10 μL at 37 °C for 30 min, heat inactivated at 75 °C for 10 min, and then subjected to cDNA synthesis, which was carried out in 20 μL volumes containing 0.1 μM of each TERRA- and 18S rRNA-specific primers and 200 U Moloney murine leukemia virus (M-MLV) reverse transcriptase (Thermo Fisher) according to the manufacturer’s instructions. To ensure that genomic DNA was completely removed, the RT reaction mixture without M-MLV reverse transcriptase was subjected to PCR with one of the TERRA primer pairs, (e.g., 10q). PCR was performed in duplicate with 1 μL cDNA template, 0.5 μM primers and 1 × LightCycler^®^ 480 SYBR Green I Master mix (Roche) by using the LightCycler^®^ 480 system (Roche). For the amplification of 18S rRNA, 1 μL cDNA dilution (200 × or 100 ×) was used. PCR was conducted at 95 °C for 5 min, and then 40 cycles of 95 °C for 15 s and 60 °C for 1 min, following a dissociation stage at 95 °C for 5 s, 65 °C for 1 min, and then 40 °C for 30 s for melting curve analysis. Sequences of primers used for RT–qPCR for TERRA detection are listed in S1 Table. The melting curve for each primer set used for TERRA detection is shown in S1 Fig. RT–qPCR for determination of gene expression was performed at the conditions required for TERRAs with slight modifications. Briefly, cDNA was synthesized from 1 μg of DNase I-treated total RNA with 6 μM random hexamers (Takara), and PCR was performed with 0.2 μM primers. Sequences of the gene-specific primers are listed in S2 Table.

### Northern blotting

Northern blotting was performed as described in Current Protocols in Molecular Biology [29], with modifications. Briefly, 10 μg of DNase I-treated total RNA was fractionated on 1.3% agarose formaldehyde gels. RNA on the gel was transferred onto a nylon membrane (GE Healthcare) by capillary transfer with 10 × SSC buffer for 3 days at room temperature by replacing the transfer buffer with a fresh one each day. Membranes were hybridized with DIG-labeled d(CCCTAA)4 at 45°C overnight. Washing and detection were performed as described in the methods for Southern blotting (above). The hybridization reaction was stripped out by washing the membrane at 80°C in freshly prepared stripping buffer (50% deionized formamide/5% SDS/50 mM Tris-HCl, pH 7.5) for 2 × 60 min, and the next rounds of hybridization were followed with a DIG-labeled d(TTAGGG)4 and then with a DIG-labeled 18S rDNA probe. Image Lab^™^ analysis software (v. 6, Bio-Rad) was used for quantification of TERRAs.

### Chromatin immunoprecipitation (ChIP) assays

ChIP assays were performed as described [30], with modifications. Cells crosslinked with formaldehyde were resuspended in buffer A (5 mM PIPES, pH 8.0, 85 mM KCl, 0.5% (v/v) Nonidet P-40) for 10 min on ice and microcentrifuged at 5000 rpm for 5 min at 4 °C to pellet the nuclei. This pellet was resuspended in SDS-lysis buffer (1% SDS, 10 mM EDTA, 50 mM Tris-HCl, pH 8.1) and sonication was followed using a Bioruptor (BMS, Seoul, Korea) at high power for 40 cycles (30 s on and 30 s off). For immunoprecipitation, 10 μg chromatin was used for the TRF2- and γ-H2AX-ChIP assays and 20 μg chromatin for RNAPII. Antibodies used were 2 μg anti-TRF2 (ab108997, Abcam, Cambridge, MA, USA), 2 μg anti-γ-H2AX (ab2893, Abcam), 3 μg anti-RNAPII pS2 (ab5095, Abcam), 4 μg anti-RNAPII pS5 (05–623, Millipore, Bedford, MA, USA), and corresponding amounts of normal rabbit IgG (sc2027, Santa Cruz Biotechnology, Dallas, TX, USA) and normal mouse IgG (12–371, Millipore). The lysates were incubated with antibodies at 4 °C overnight and supplemented with 50 μL protein A/G agarose-PLUS (Santa Cruz Biotechnology) at 4 °C for 90 min. Immune complexes were washed and eluted as recommended by the EZ ChIP^™^ user’s guide (Millipore). For slot blotting, DNA was isolated using PCR purification kits (Nucleogen, Seoul, Korea), heat-denatured, and loaded on slot (70% for telomere detection, 25% for centromere detection). The slot blot was UV-crosslinked and immersed in denaturing buffer (1.5 M NaCl, 0.5 M NaOH) for 15 min, and then in neutralization buffer (1.5 M NaCl, 0.5 M Tris-HCl, pH 7.4) for 10 min. After rinsing the blot with water, hybridization and detection was performed as described as in the methods for Southern blotting above. A 3′–DIG-labeled d(TTAGGG)4 probe and a 3′–DIG-labeled oligonucleotide probe corresponding to the alphoid element (5′–GTTTTGAAACACTCTTTTTGTAGAATCTGC–3′) [31] was used for the detection of telomeres and centromeres, respectively. For ChIP–qPCR, RNAPII–ChIP DNA isolated by column purification was used as a template for qPCR. PCR data were normalized to input which was isolated in parallel for each experiment. Primer sequences used for ChIP–qPCR are listed in S3 Table.

## Western blot analysis

Whole-cell lysates were prepared in RIPA buffer (50 mM Tris⋅HCl, pH 8.0/150 mM NaCl/1% (v/v) Nonidet P-40/0.5% (w/v) sodium desoxycholate/0.1% (w/v) SDS) containing 1 × protease inhibitor cocktail (Roche) and resolved using SDS–PAGE. Proteins were transferred onto PVDF membranes (Immobilon^®^-P, Millipore) and detected using ChemiDoc XRS+ (Bio-Rad). Primary antibodies used were rabbit anti-TRF2 (1:1,000, ab108997, Abcam), mouse anti-γ-H2AX (1:1,000, JBW301, Millipore), rabbit anti-β-actin (1:5,000, #4967, Cell Signaling Technology, Danvers, MA, USA), and horseradish peroxidase-conjugated mouse IgG (1:5,000, sc-2005; Santa Cruz Biotechnology) and horseradish peroxidase-conjugated rabbit IgG (1:5,000, sc-2313, Santa Cruz Biotechnology) as the secondary antibodies.

### Statistics

Statistical analyses were performed using IBM SPSS Statistics (v. 24, IBM Corp., Armonk, NY, USA) and assessed using Student’s *t*-test, Mann-Whitney *U*-test, and one-way analysis of variance (ANOVA). The level of statistical significance was set at *P* < 0.05.

## Results

### Etoposide promotes telomere damage and increases the levels of TERRAs

**Fig 1:**
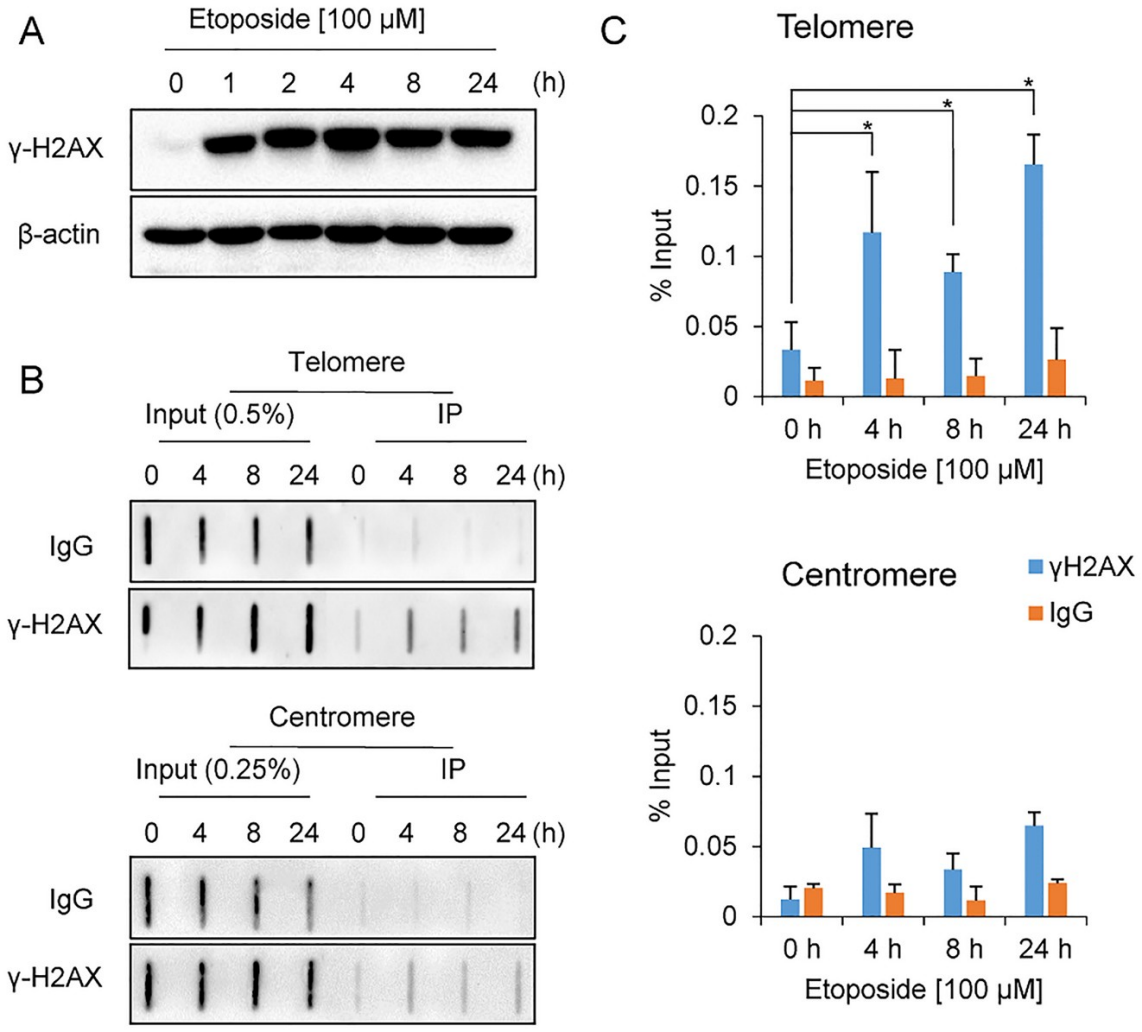
Induction of telomere damage upon etoposide treatment. A. Immunoblot of γ-H2AX in HeLa cells treated with etoposide for the indicated times; β-actin was used as a loading control. B. ChIP results for telomeric and centromeric DNA with the anti-γ-H2AX antibody in HeLa cells treated with etoposide for the indicated times. DNA slot blots were hybridized with telomere- or centromere-specific probes. Antibodies specific for γ-H2AX and control IgG were used for the ChIP assays. C. Quantification of three independent ChIP assays represented in B (mean ± standard deviation, SD). Mann-Whitney *U*-test was used to compare differences in DNA levels between each time point and 0 h; *P < 0.05.

Etoposide stabilizes the covalent DNA–topoisomerase II complex and induces DNA damage [32]. To evaluate the occurrence of DNA damage after exposure to etoposide, the DNA damage marker protein γ-H2AX was detected in HeLa cells treated with etoposide for various times. Immunoblotting showed that this marker protein was induced from the early time points of treatment (Fig 1A). Next, telomere damage was evaluated in the drug-treated cells by performing ChIP experiments using an antibody against γ-H2AX. As a result, accumulation of γ-H2AX at telomeres was observed from early time points of etoposide treatment (Fig 1B and 1C). In addition, cell viability was checked using MTT assays, which showed that etoposide treatment led to growth arrest, but cell death was not evident until 24 h (S2 Fig), so in this study, etoposide treatment was applied for up to 24 h.

**Fig 2:**
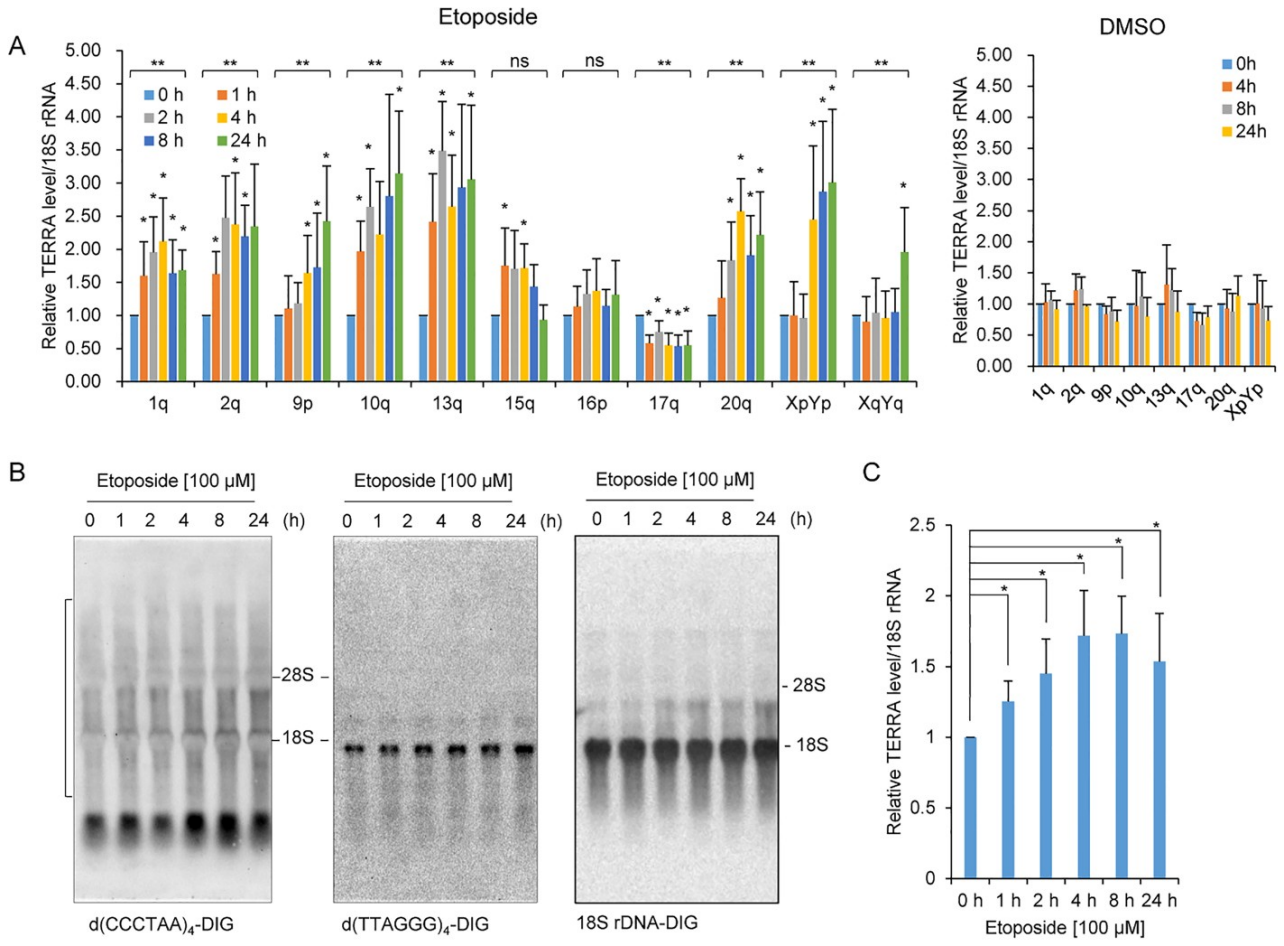
Increased levels of TERRAs in etoposide-treated HeLa cells. A. RT–qPCR analysis of TERRAs. HeLa cells were treated with 100 μM of etoposide or vehicle alone (DMSO) for the indicated times. TERRAs were measured using RT–qPCR with 11 pairs of subtelomere-specific primers as indicated. The levels of TERRAs were normalized to 18S rRNA and compared with 0 h. Error bars are SD derived from at least three independent assays. Mann-Whitney *U*-test was used to compare TERRA levels between each time point and 0 h; an asterisk (*) on the bars denotes *P* < 0.05. One-way ANOVA was used for comparing TERRA levels at various incubation times for each representative subtelomere; **P < 0.05, and ns *P* ≥ 0.05. B. Northern blot analysis of TERRAs. Total RNAs were isolated from HeLa cells treated with etoposide for the indicated times. The DIG-labeled probes used in hybridization are indicated. The membrane was reprobed after stripping off the previous probes. Size markers, 28S and 18S rRNAs, are indicated. The d(CCCTAA)4-DIG-specific diffuse bands, indicated by a bracket, were quantified, normalized to 18S bands, and compared with 0 h. C. Quantification of northern blot analyses in B (mean ± SD; *n* = 4). One-way ANOVA was used for comparing TERRA levels according to different incubation times (*P* = 0.004). Mann-Whitney *U*-test was used to compare differences in TERRA levels between each time point and 0 h; *P < 0.05.

Next, we investigated whether DNA damage would affect the regulation of TERRAs. To do so, the levels of TERRAs were measured by RT–qPCR with 11 pairs of subtelomere-specific primers in HeLa cells treated with etoposide for various times (S1 Table and S1 Fig). Note that many of the primers used for TERRA detection targeted various subtelomeres because of sequence degeneration at subtelomeres (S1 Table). In this study, we will refer to TERRA amplified with the subtelomere primer pairs, e.g., 1q primers, as TERRA-1q. Etoposide treatment led to a progressive increase in the levels of TERRAs amplified with the primers specific to 1q, 2q, 9p, 10q, 13q, 20q, XpYp in a time-dependent manner (Fig 2A). The time-dependent increase was less obvious for TERRA-15q, -16p, -17q, or -XqYq. Nonetheless, increased levels were detected in TERRA-15q at early times of treatment and TERRA-XqYq at 24 h, compared with 0 h (Fig 2A). On the other hand, the levels of TERRA-17q decreased after exposure to etoposide while no significant changes were found in TERRA-16p. RT–qPCR of TERRAs in DMSO (vehicle alone)-treated cells showed that DMSO had no effect on TERRA levels (Fig 2A). Collectively, etoposide treatment resulted in induction of TERRAs stemming from various chromosome ends, including 20q and XpYp, whereas several TERRAs did not undergo distinct upregulation upon etoposide treatment.

We checked the total levels of TERRAs in time-course experiments by northern blotting. Hybridization with a strand-specific TERRA probe, 3′–DIG-labeled d(CCCTAA)4, showed diffuse bands, which are typical TERRA features. The same membrane stripped and hybridized with the DIG-labeled d(TTAGGG)4 probe showed a distinct band near 18S rRNA and verified that signals from the first hybridization had been erased (Fig 2B). Quantification of the d(CCCTAA)4 probe-specific smear bands revealed increased TERRAs in etoposide-treated cells, compared with 0 h, (Fig 2C). Gradual increases of TERRAs were observed in time-dependent manners (Fig 2C), which was somewhat consistent with the results of RT–qPCR of TERRAs (Fig 2A).

### No changes in telomere length or TRF2 abundance were found at telomeres upon etoposide treatment

**Fig 3:**
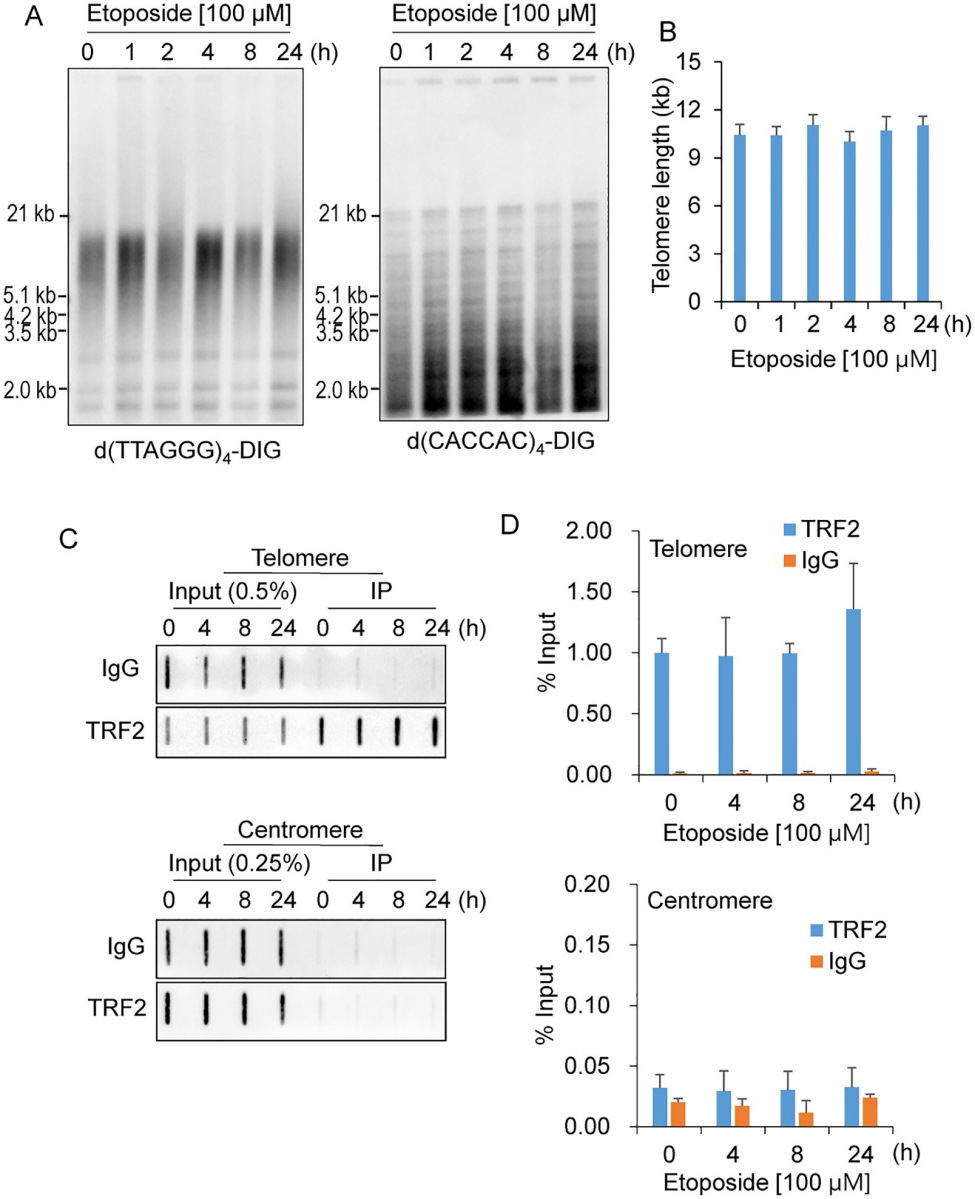
Telomere length and TRF2-mediated telomere integrity were maintained in etoposide-treated HeLa cells. A. Southern blot analysis of telomeres. Terminal restriction fragments were detected in HeLa cells treated with etoposide for the indicated times. The blot was hybridized with a DIG-labeled d(TTAGGG)4 probe (left panel), stripped, and then rehybridized with a DIG-labeled d(CACCAC)4 probe (right panel). Size markers are indicated on the left. B. Telomere length in HeLa cells treated with etoposide. The telomere lengths were determined as described previously [27], and the mean telomere length was obtained from two independent experiments (mean ± SD). C. ChIP assays in HeLa treated with etoposide for the indicated hours. DNA precipitates were slot-blotted and hybridized with telomeric and alphoid repeat-containing probes. Antibodies specific for TRF2 and control IgG were used for the ChIP assays. D. Quantification of ChIP assays represented in C (mean ± SD; *n* = 3).

To investigate whether etoposide-induced telomere damage was a result of shortened telomeres, terminal restriction fragment lengths were measured using Southern blotting in HeLa cells treated with etoposide for various times. Hybridization with the telomere-specific 3′–DIG-labeled d(TTAGGG)4 probe revealed that exposure of cells to etoposide did not lead to shortening of telomeres (Fig 3A and 3B). The same membranes stripped and hybridized to a 3′–DIG-labeled d(CAC)8 probe for minisatellite DNA showed that genome integrity was maintained during the course of incubation (Fig 3A).

Impairment of telomere-protective factors leads to telomere damage [2]. In fact, Porro *et al*. showed that zeocin-mediated telomere damage was attributed to TRF2 loss at telomeres [19]. To address this possibility, we performed ChIP assays with an antibody against TRF2 in HeLa cells treated with etoposide for 0, 4, 8, or 24 h. These showed that the occupancy of TRF2 at telomeres remained unchanged during the course of incubation (Fig 3C and 3D). This protein was barely detected at centromeres under any condition, confirming that TRF2-binding to telomeres is specific (Fig 3C). Taken together, because telomere length and TRF2-mediated telomere integrity were maintained under our experimental conditions, these two factors are unlikely to be relevant to telomere damage or upregulation of TERRAs in response to DNA damage.

Previous studies revealed that etoposide treatment results in varied expression of telomere-related genes such as those encoding TRF2 and telomerase [33,34]. In fact, we noticed elevated TRF2 protein levels at later incubation times (A in S3 Fig) although its mRNA was not significantly upregulated at those times (B in S3 Fig). This result is somewhat in agreement with a report that showed increased TRF2 gene expression in etoposide-treated HL60 leukemia cells [33]. We further analyzed RNA levels of telomere-related genes such as *TRF1*, encoding one member of the shelterin complex, *TERT*, encoding a catalytic component of telomerase, and *TERC*, an RNA component of telomerase (B in S3 Fig). The RNA levels of *TRF1* and *TERT* decreased at 24 h, whereas *TERC* expression remained unchanged during incubation (B in S3 Fig). Whether regulation of these telomere-related gene expressions is important in cellular responses to DNA damage remains elusive.

### Increased stability of TERRAs upon DNA damage

**Fig 4:**
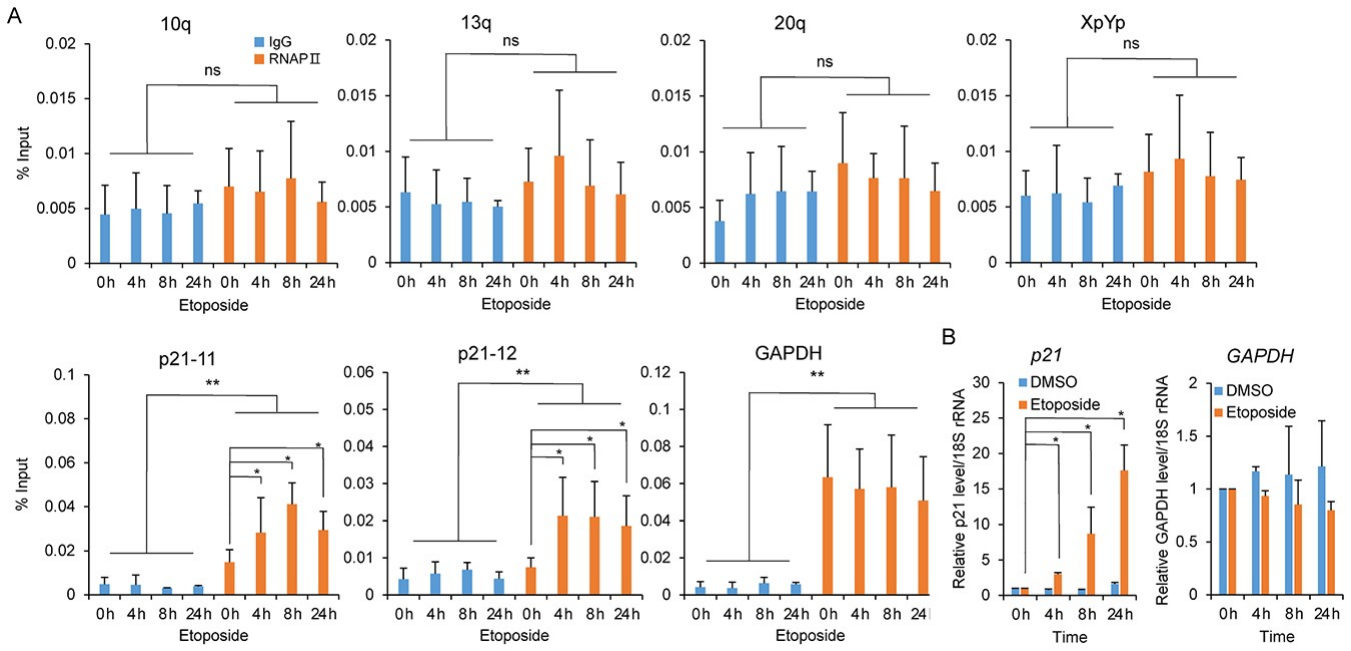
ChIP–qPCR to assay RNAPII binding at subtelomeres after exposure to etoposide. HeLa cells incubated with etoposide at 100 μM for the indicated times were subjected to ChIP assays using an anti-phosphorylated RNAPII antibody (pS2) and IgG as a control. Associated DNA fragments were PCR-amplified for p21, GAPDH, and subtelomeres as indicated; p21-11 and p21-12 indicate the p21 gene at 0.2 and 2.1 kb from the transcription start site, respectively. Results are presented as percentages of input DNA. Bars represent the SDs of at least three replicates. Student’s *t*-test was used to compare DNA levels between RNAPII-ChIP and IgG-ChIP; **P < 0.05 and ns indicates *P* ≥ 0.05. Mann-Whitney *U*-test was used to compare DNA levels between each h and 0 h; *P < 0.05. B. RT–qPCR for *p21* and *GAPDH* in HeLa cells treated with DMSO (vehicle alone) or etoposide at 100 μM for the indicated times. *p21* and *GAPDH* levels were normalized to 18S rRNA and expressed as fold changes relative to 0 h. Mann-Whitney *U*-test was used to compare DNA levels between each h and 0 h; *P < 0.05.

We wondered whether upregulation of TERRAs might be associated with increased transcriptional activity at telomeres. To address this issue, we performed ChIP experiments in HeLa cells treated with etoposide for 0, 4, 8, and 24 h using an antibody against the elongating form of RNAPII (pS2; Fig 4A). As a control, the occupancy of RNAPII on the p21 gene locus was evaluated using qPCR (Fig 4A). Genotoxic stress is known to induce p21, a cyclin-dependent kinase inhibitor that plays an important role in preventing cell cycle progression [35,36]. Indeed, our RT–qPCR results verified p21 upregulation following exposure to etoposide (Fig 4B). ChIP–qPCR experiments revealed that etoposide treatment led to the accumulation of RNAPII at the p21 transcription regions, in particular, a time-dependent increase of RNAPII binding at 0.2 kb from the transcription start site (p21-11, one-way analysis of variance, ANOVA, *P* = 0.015, Fig 4A). These indicated that etoposide-mediated p21 induction was associated with enhanced transcriptional activity. We also checked the GAPDH gene locus in the ChIP DNA precipitates and found enrichment of RNAPII on the GAPDH gene regardless of etoposide treatment (Fig 4A), which reflects a constitutive expression of this gene under any conditions (Fig 4B). The binding of RNAPII to the subtelomeres for 10q, 13q, 20q, and XpYp were evaluated. However, we found no significant enrichment of RNAPII at any of these subtelomeres during the course of incubation with etoposide (Fig 4A). In fact, binding of RNAPII to the subtelomeres was very weak: nearly at the levels of IgG binding (Fig 4A). We further performed a ChIP–slot assay to find the interaction of RNAPII with telomeres (S4 Fig). This showed a specific interaction of RNAPII with telomeres but revealed no significant differences in telomere binding of RNAPII with various incubation times with etoposide (A and B in S4 Fig) A similar result was observed when using an antibody against the initiation form (RNAPII pS5; C, D in S4 Fig). These findings may suggest that transcription rates *per se* do not account for the accumulation of TERRAs.

**Fig 5:**
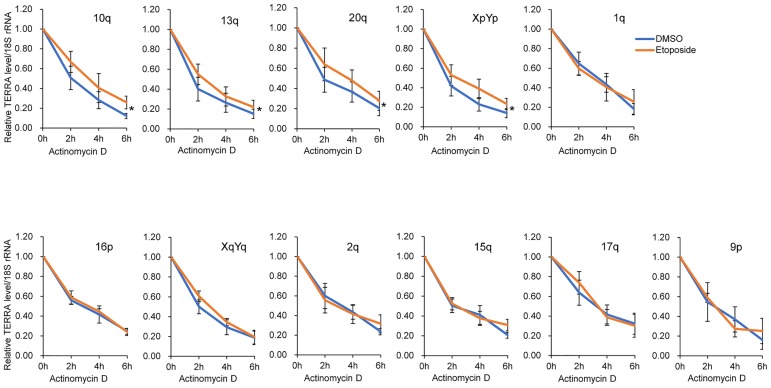
Stability of TERRAs in etoposide-treated cells. HeLa cells incubated with DMSO (vehicle alone) or etoposide at 100 μM for 24 h were treated with actinomycin D at 5 μg/mL for the indicated times. TERRAs were measured using RT–qPCR with primers as indicated, normalized against 18S rRNA, and compared with 0 h. Error bars are SDs derived from at least five independent experiments. Student’s *t*-tests were used to compare differences in TERRA levels between etoposide- and DMSO-treated cells; *P < 0.05.

To assess whether the induction of TERRAs might be a result of increased RNA stability, we blocked de novo gene transcription in etoposide- or DMSO-treated HeLa cells with actinomycin D and monitored the levels of TERRAs in a time-course experiment. Stability of TERRAs was evaluated with all subtelomere primer pairs. Interestingly, TERRA-10q, -13q, -20q, and -XpYp degraded at a slower rate in etoposide-treated cells than in mock-treated controls (Fig 5). In contrast, decay rates of the other TERRAs were nearly identical in both cells (Fig 5). These findings suggest that the accumulation of certain TERRAs can be attributed, at least in part, to RNA stabilization. As controls, the stabilities of *c-myc*, *GAPDH*, and *β-actin* were evaluated (S5 Fig). These genes showed different patterns of mRNA stability. For instance, degradation of *c-myc* mRNA was rapid and slowed in etoposide-treated cells, whereas *GAPDH* and *β-actin* were stable in both etoposide- and mock-treated cells (S5 Fig).

## Discussion

In this study, we measured the levels of TERRAs using RT–qPCR and found that exposure of cells to etoposide led to progressive time-dependent increases in the levels of TERRAs. Northern blot analysis of total TERRA also showed increased levels of TERRAs in etoposide-treated cells. We employed 11 pairs of primers targeting subtelomere sequences in various chromosomes, and the results showed that TERRAs from not only the 20q and XpYp loci but also many other chromosomal loci accumulated upon DNA damage. However, this induction was not evident in several TERRAs: for instance, the levels of TERRA-17q decreased while there were no obvious changes in TERRA-16p after exposure to etoposide. Montero *et al*. reported that transcripts arising from the subtelomeres of chromosomes 1p, 9p, 12p, 15q, 16p, 19p, and XqYq are unlikely to be true TERRAs and that telomere transcripts are generated mainly from the 20q and XpYp loci [13]. These findings are unexpected because it has been widely accepted that transcription occurs at many chromosome terminals [19–21]. In our study, the primers for 9p, 15q, 16p, and XqYq targeted the subtelomeres of undesignated chromosome loci (S1 Table). Nevertheless, etoposide-mediated induction was observed for TERRA-9p while it was less obvious for the other three. Further study is needed to characterize telomere transcription in detail. In particular, whether telomere transcripts generated from many other chromosome loci are TERRAs or TERRA-like transcripts remains to be elucidated.

In a previous report, zeocin treatment resulted in the induction of TERRAs, which was accompanied by TRF2 loss at telomeres [19]. TRF2 is known to suppress transcription of TERRAs directly through its TRFH domain [19]. However, this was not the case for etoposide-induced upregulation of TERRAs. Etoposide promoted telomere damage as revealed by the γ-H2AX–ChIP assays but the abundance of TRF2 at telomeres remained unaltered. Etoposide is known to increase topoisomerase II-mediated DNA breakage by inhibiting its ability to religate cleaved nucleic acid molecules [32,37]. Nonetheless, telomere shortening during the course of incubation was not evident in our study. Therefore, we speculate that actual cleavage did not occur under our experimental conditions. Because telomere lengths and TRF2 occupancy at telomeres were not altered, the nature of etoposide-mediated telomere damage remains to be elucidated.

Association of RNAPII at the subtelomeres of 10q, 13q, 20q, and XpYp was very weak at background levels but abundance of the enzyme at telomeres was evident as revealed by the RNAPII ChIP-slot assays. Nonetheless, there were no significant changes in the telomeric or subtelomeric binding activity of RNAPII in response to DNA damage. Interestingly, RNA stability assays in the presence of actinomycin D revealed slower degradation of TERRA-20q, -XpYp, -10q, and -13q in etoposide-treated cells. These suggest that accumulation of these TERRAs can be attributed to increased RNA stability. We observed an increase of TRF2 protein levels at later times of etoposide treatment. Since TRF2 binds TERRA [16], it is possible that the amount of TERRA-bound TRF2 is increased, and this could contribute to the increased TERRA stability. Azzalin and Lingner showed that a small fraction of TERRAs—around 7%—have poly A tails [9]. Poly A^+^ TERRA was found to have a long half-life compared with poly A^−^ TERRA [38]. Whether increased stability of TERRAs is conferred by the presence of poly A^+^ TERRA in a larger fraction or via another protective mechanism, such as suppression of RNA degradation, remains elusive.

Meanwhile, TERRAs from many loci tested in this study were degraded at similar rates in both drug- and mock-treated cells. Currently, we do not fully understand the mechanism of etoposide-mediated induction of these TERRAs. The results of RNAPII ChIP-slot assay suggested that global telomere transcription does not vary during etoposide treatment. It is not clear if this applies to each single telomere. A recent work from Feretzaki *et al*. showed that the absolute levels of TERRA from different telomeres vary significantly: TERRA 17p is very abundant, while TERRA 2p is barely detected in HeLa cells [21], suggesting that each telomere may be transcribed at different levels by RNAPII. This raises the possibility that global RNAPII distribution at telomeres may not reflect RNAPII interaction at each single telomere. Unfortunately, the RNAPII ChIP at single telomere did not work in our system, and in fact this method had been successful in U2OS (osteosarcoma, an ALT cell line) and HCT116 (a human colon cancer cell line) cells [20], which express high levels of TERRAs. It was reported that TERRA is much more abundant in U2OS cells than in HeLa cells, ranging from 4- to 80-fold high depending on the subtelomeres [21]. HeLa cells might not be suitable for the RNAPII ChIP at single telomere because of a weak transcriptional activity at the region. Further studies on U2OS cells may provide more comprehensive understanding of TERRA regulation at the transcriptional level in response to DNA damage. The results of RT-qPCR for TERRA showed that induction of some TERRAs following etoposide treatment was very fast. For instance, TERRA-10q and -13q levels double after 1 h (Fig 2A), while half-lives of these TERRAs were in the order of 2–3 h (Fig 5). A rapid increase in these TERRAs could be attributed partly to enhanced transcriptional activity. Therefore, we do not rule out the possibility that transcriptional regulation could be part of the mechanism that leads to TERRA induction following etoposide treatment.

In summary, etoposide treatment led to progressive time-dependent increases in TERRAs, and this was detected at many chromosome loci but not all. Telomere damage occurred from early times of etoposide treatment but telomere lengths and TRF2-mediated telomere integrity were maintained during the course of incubation. Transcriptional activity at telomeres in response to DNA damage remained unchanged. The accumulation of TERRAs from certain loci including 20q and XpYp could be attributed to RNA stabilization. Our study may provide insight into the post-transcriptional regulation of TERRAs in response to DNA damage.

## Supporting information

S1 Table List of primers used for RT-qPCR for TERRA detection. (DOCX)

S2 Table List of primers used for RT-qPCR of gene expression. (DOCX)

S3 Table List of primers used for ChIP-qPCR. (DOCX)

S1 Fig Melting curve analysis from RT–qPCR of TERRAs. Dissociation curves for TERRAs transcribed from subtelomeres at various chromosomal loci. cDNA replaced with PCR-grade water was used as the no-template control, and this revealed no peak.
(TIFF)

S2 Fig Cell viability upon treatment with etoposide. MTT assays were performed on HeLa cells treated with etoposide at 100 μM for the indicated times and cell viability was measured relative to 0 h. Error bars are based on three independent experiments (mean ± SD).
(TIFF)

S3 Fig Expression of telomere-related genes in etoposide-treated cells. A. Immunoblot for TRF2 in etoposide-treated HeLa cells. Whole-cell lysates prepared from HeLa cells treated with etoposide at 100 μM for the indicated times were subjected to SDS–PAGE followed by immunoblotting. The β-actin was used as a loading control. B. RT–qPCR for telomere-related genes in etoposide-treated HeLa cells. RT–qPCR was performed using HeLa cells treated with etoposide at 100 μM for the indicated times. The gene levels were normalized to 18S rRNA and expressed as fold changes relative to 0 h. Error bars are based on three independent experiments (mean ± SD). Mann-Whitney *U*-test was used to compare RNA levels between each h and 0 h; *P < 0.05.
(TIFF)

S4 Fig Absence of significant changes in telomeric binding of RNAPII after exposure to etoposide. ChIP–slot assays of telomeric and centromeric DNA with an anti-RNAPII antibody and IgG as a control were performed in HeLa cells incubated with etoposide at 100 μM for the indicated times. DNA precipitates were slot-blotted and hybridized with telomeric and centromeric probes. A. ChIP–slot assay using an anti-RNAPII (pS2) antibody. B. Quantification of ChIP–slot assays represented in A (mean ± SD; *n* = 3). C. ChIP–slot assay using an anti-RNAPII (pS5) antibody. D. Quantification of ChIP–slot assays represented in C (mean ± SD; *n* = 3). Student’s *t*-tests were used to compare differences in DNA levels between RNAPII-ChIP and IgG-ChIP; *P < 0.05 and ns indicates *P* ≥ 0.05.
(TIFF)

S5 Fig Stability of *GAPDH*, *β-actin*, and *c-myc* in etoposide-treated cells. HeLa cells incubated with DMSO (vehicle alone) or etoposide at 100 μM for 24 h were treated with actinomycin D at 5 μg/mL for the indicated times. *GAPDH*, *β-actin*, and *c-myc* were measured by RT–qPCR, normalized against 18S rRNA, and compared with 0 h. Error bars are derived from three independent experiments (mean ± SD). Student’s *t*-test was used to compare differences in gene levels between etoposide- and DMSO-treated cells; *P < 0.05 and ns indicates *P* ≥ 0.05.
(TIFF)
