# Supplementary material for: Increased amounts and stability of telomeric repeat-containing RNA (TERRA) following DNA damage induced by etoposide
Source: PLoS One. 2019 Nov 22;14(11):e0225302. doi: 10.1371/journal.pone.0225302 (PMC6874320; doi:10.1371/journal.pone.0225302)
Supplement: S1 Table — (DOCX) [file pone.0225302.s001.docx]

**S1 Table. List of primers used for RT-qPCR for TERRA detection.**

| Primer | Sequence (5’-3’) | Known Loci | References |
| --- | --- | --- | --- |
| TERRA-specific RT-primer | CCCTAACCCTAACCCTAACCCTAACCCTAA | N/A | 28 |
| 18S-specific RT-primer | TCCCAAGATCCAACTACGAG | N/A |  |
| 1q TERRA - forward | GCATTCCTAATGCACACATGAC | 1q | 28 |
| 1q TERRA - reverse | ACCCTAACCCGAACCCTA |  |  |
| 2q TERRA - forward | AAAGCGGGAAACGAAAAGC | 1q, 2q, 4q, 10q, 13q, 21q, 22q | 20, 28 |
| 2q TERRA - reverse | GCCTTGCCTTGGGAGAATCT |  |  |
| 9p TERRA - forward | GAGATTCTCCCAAGGCAAGG | 9p, XqYq | 28 |
| 9p TERRA – reverse | ACATGAGGAATGTGGGTGTTAT |  |  |
| 10q TERRA – forward | GAATCCTGCGCACCGAGAT | 2q, 4q, 10q, 13q | 38 |
| 10q TERRA – reverse | CTGCACTTGAACCCTGCAATAC |  |  |
| 13q TERRA – forward | CCTGCGCACCGAGATTCT | 2q, 4q, 10q, 13q | 20, 28 |
| 13q TERRA – reverse | GCACTTGAACCCTGCAATACAG |  |  |
| 15q TERRA – forward | CAGCGAGATTCTCCCAAGCTAAG | 15q | 28, 38 |
| 15q TERRA - reverse | AACCCTAACCACATGAGCAACG |  |  |
| 16p TERRA – forward | TGCAACCGGGAAAGATTTTATT | 8p, 16p | 20 |
| 16p TERRA – reverse | GCCTGGCTTTGGGACAACT |  |  |
| 17q TERRA – forward | GTCCATGCATTCTCCATTGATAAG | 17q | 20, 28 |
| 17q TERRA – reverse | AGCTACCTCTCTCAACACCAAGAAG |  |  |
| 20q TERRA – forward | ACATGGGCGATACTCAGG | 20q | 13 |
| 20q TERRA – reverse | CCCACTACTGTGCCTCAA |  |  |
| XpYp TERRA - forward | AAGAACGAAGCTTCCACAGTAT | XpYp | 28 |
| XpYp TERRA – reverse | GGTGGGAGCAGATTAGAGAATAAA |  |  |
| XqYq TERRA – forward | GAAAGCAAAAGCCCCTCTGA | 9p, XqYq | 20, 28 |
| XqYq TERRA - reverse | CCCCTTGCCTTGGGAGAA |  |  |
| 18S – forward | CATTGGAGGGCAAGTCTGG | N/A |  |
| 18S - reverse | TCCCAAGATCCAACTACGAGC |  |  |

N/A, not applicable
