# Supplementary material for: Increased amounts and stability of telomeric repeat-containing RNA (TERRA) following DNA damage induced by etoposide
Source: PLoS One. 2019 Nov 22;14(11):e0225302. doi: 10.1371/journal.pone.0225302 (PMC6874320; doi:10.1371/journal.pone.0225302)
Supplement: S2 Table — (DOCX) [file pone.0225302.s002.docx]

**S2 Table. List of primers used for RT-qPCR of gene expression.**

| Primer | Sequence (5’-3’) | References |
| --- | --- | --- |
| TRF1 - forward | TGCTTTCAGTGGCTCTTCTG | 39 |
| TRF1 - reverse | ATGGAACCCAGCAACAAGAC | 39 |
| TRF2 - forward | TTGTGGGGTCCTTGGACATA | 39 |
| TRF2 - reverse | CCAGTAGAAAACTGGTCAAGGAA | 39 |
| TERT - forward | TGTGCACCAACATCTACAAG | 40 |
| TERT - reverse | GCGTTCTTGGCTTTCAGGAT | 40 |
| TERC - forward | TCTAACCCTAACTGAGAAGGGCGTAG | 41 |
| TERC - reverse | GTTTGCTCTAGAATGAACGGTGGAAG | 41 |
| β-actin - forward | TGTACGCCAACACAGTGCTG | 42 |
| β-actin - reverse | GCTGGAAGGTGGACAGCGA | 42 |
| GAPDH - forward | AGCCACATCGCTCAGACAC | 20 |
| GAPDH - reverse | GCCCAATACGACCAAATCC | 20 |
| 18S - forward | CATTGGAGGGCAAGTCTGG | 43 |
| 18S - reverse | TCCCAAGATCCAACTACGAGC | 43 |
| c-Myc - forward | ACTCTGAGGAGGAACAAGAA | 40 |
| c-Myc - reverse | TGGAGACGTGGCACCTCTT | 40 |
| p21 - forward | ACCATGTGGACCTCTCACTGT | 44 |
| p21 - reverse | TTAGGGCTTCCTCTTGGAGAA | 44 |

References in this table:

1. Scheibe M, Arnoult N, Kappei D, Buchholz F, Decottignies A, Butter F, et al. [Quantitative interaction screen of telomeric repeat-containing RNA reveals novel TERRA regulators.](https://www.ncbi.nlm.nih.gov/pubmed/23921659) Genome Res. 2013;23: 2149–2157.
2. Park IH, Zhao R, West JA, Yabuuchi A, Huo H, Ince TA, et al. [Reprogramming of human somatic cells to pluripotency with defined factors.](https://www.ncbi.nlm.nih.gov/pubmed/18157115) Nature. 2008;451: 141–146.
3. Lundberg AS, Randell SH, Stewart SA, Elenbaas B, Hartwell KA, Brooks MW, et al. [Immortalization and transformation of primary human airway epithelial cells by gene transfer.](https://www.ncbi.nlm.nih.gov/pubmed/12085236) Oncogene. 2002;21: 4577–4586.
4. Arnoult N, Van Beneden A, Decottignies A. [Telomere length regulates TERRA levels through increased trimethylation of telomeric H3K9 and HP1α.](https://www.ncbi.nlm.nih.gov/pubmed/22922742) Nat Struct Mol Biol. 2012;19: 948–956.
5. Saunus JM, Edwards SL, French JD, Smart CE, Brown MA. Regulation of *BRCA1* messenger RNA stability in human epithelial cell lines and during cell cycle progression. FEBS Lett. 2007;581: 3435–3442.
6. Liu R, Wang L, Chen G, Katoh H, Chen C, Liu Y, et al. FOXP3 up-regulates p21 expression by site-specific inhibition of histone deacetylase 2/histone deacetylase 4 association to the locus. Cancer Res. 2009;69: 2252–2259.

Reference no. 20 is listed in the main References section.
