# Supplementary material for: Increased amounts and stability of telomeric repeat-containing RNA (TERRA) following DNA damage induced by etoposide
Source: PLoS One. 2019 Nov 22;14(11):e0225302. doi: 10.1371/journal.pone.0225302 (PMC6874320; doi:10.1371/journal.pone.0225302)
Supplement: S3 Table — (DOCX) [file pone.0225302.s003.docx]

**S3 Table. List of primers used for ChIP-qPCR.**

| Primer | Sequence (5’-3’) | References | |
| --- | --- | --- | --- |
| GAPDH exon 1 - forward | CTCCTGTTCGACAGTCAGC | | 45 |
| GAPDH exon 1- reverse | TTCAGGCCGTCCCTAGC | | 45 |
| p21 ChIP 11 - forward | AGGCACTCAGAGGAGGTGAGA | | 44 |
| p21 ChIP 11- reverse | CAGAAACACCTGTGAACGCA | | 44 |
| p21 ChIP 12 - forward | TAAGGCAGGAAGGCCAATAA | | 44 |
| p21 ChIP 12- reverse | TCTCCTCCCCCATCAAAAT | | 44 |
| 10q TERRA – forward | GAATCCTGCGCACCGAGAT | | 38 |
| 10q TERRA – reverse | CTGCACTTGAACCCTGCAATAC | | 38 |
| 13q TERRA – forward | CCTGCGCACCGAGATTCT | | 20, 28 |
| 13q TERRA – reverse | GCACTTGAACCCTGCAATACAG | | 20, 28 |
| 20q TERRA – forward | ACATGGGCGATACTCAGG | | 13 |
| 20q TERRA – reverse | CCCACTACTGTGCCTCAA | | 13 |
| XpYp TERRA - forward | AAGAACGAAGCTTCCACAGTAT | | 28 |
| XpYp TERRA – reverse | GGTGGGAGCAGATTAGAGAATAAA | | 28 |

References in this table:

1. Liu R, Wang L, Chen G, Katoh H, Chen C, Liu Y, et al. FOXP3 up-regulates p21 expression by site-specific inhibition of histone deacetylase 2/histone deacetylase 4 association to the locus. Cancer Res. 2009;69: 2252–2259.
2. Glover-Cutter K, Kim S, Espinosa J, Bentley DL. RNA polymerase II pauses and associates with pre-mRNA processing factors at both ends of genes. Nat Struct Mol Biol. 2008;15: 71–78.

Other references are listed in the main References section.
