# Supplementary figures and images for: Increased amounts and stability of telomeric repeat-containing RNA (TERRA) following DNA damage induced by etoposide
Source: PLoS One. 2019 Nov 22;14(11):e0225302. doi: 10.1371/journal.pone.0225302 (PMC6874320; doi:10.1371/journal.pone.0225302)

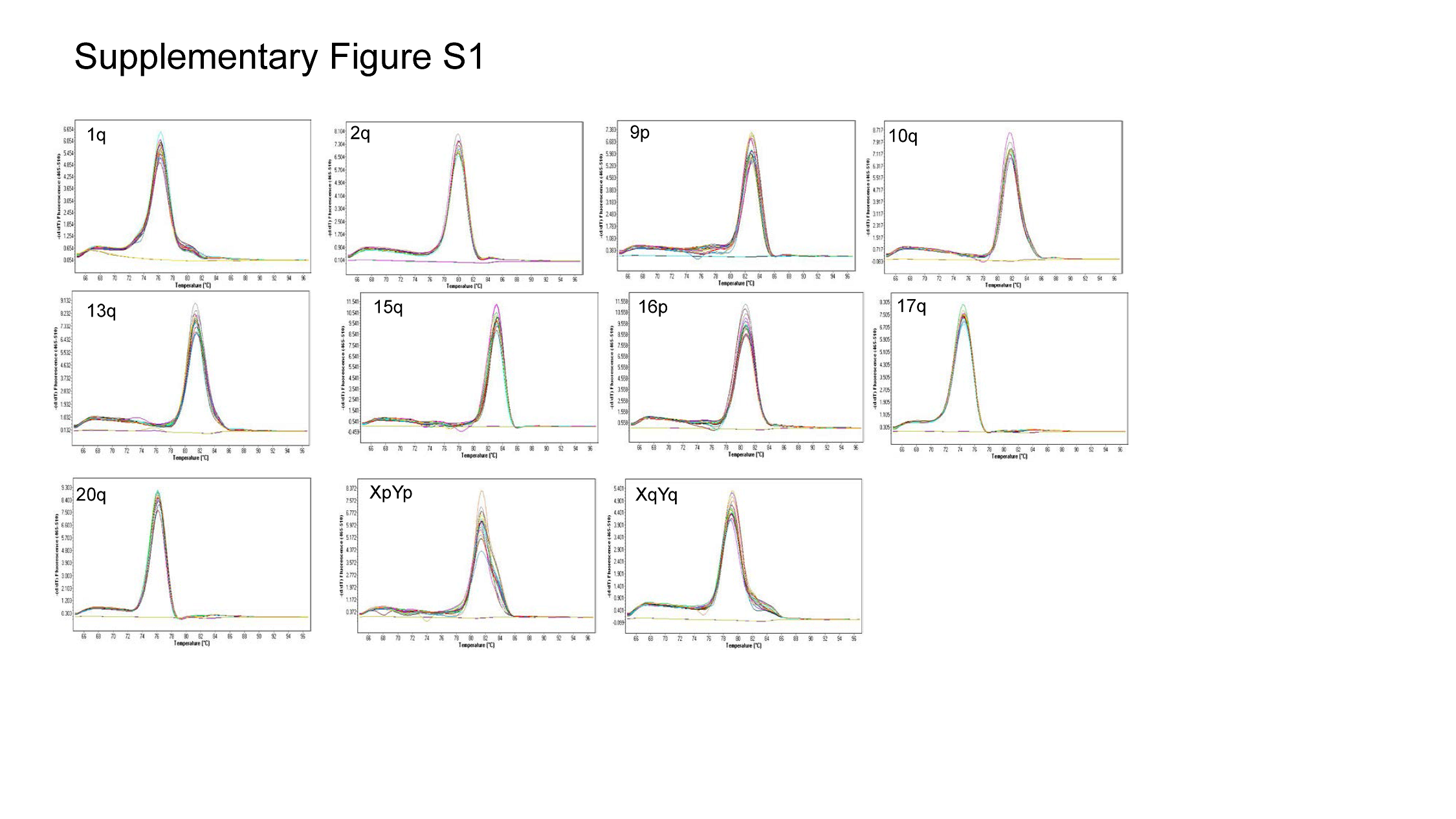

Supplement: S1 Fig — Dissociation curves for TERRAs transcribed from subtelomeres at various chromosomal loci. cDNA replaced with PCR-grade water was used as the no-template control, and this revealed no peak. (TIFF) [file pone.0225302.s004.tiff]

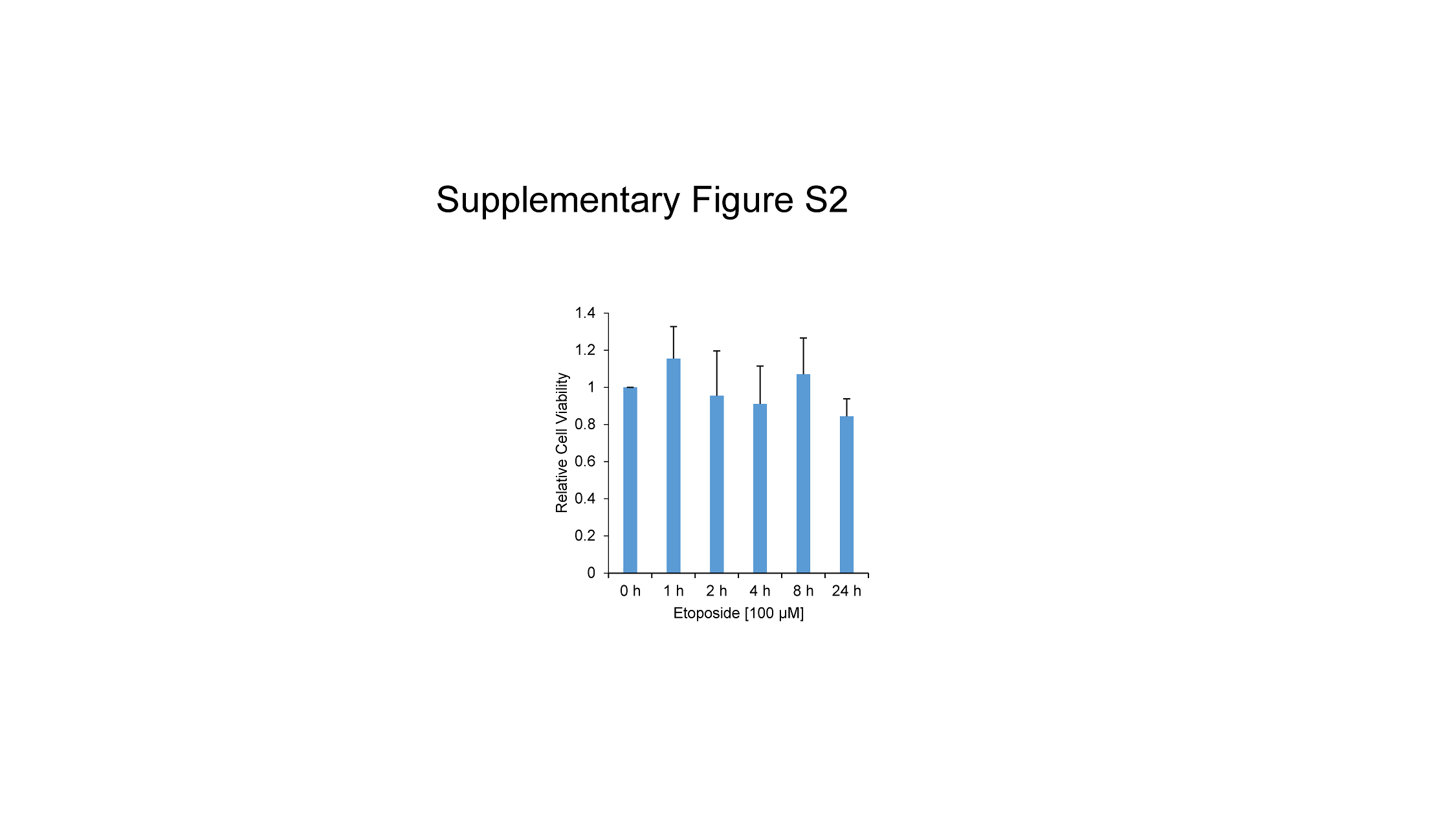

Supplement: S2 Fig — MTT assays were performed on HeLa cells treated with etoposide at 100 μM for the indicated times and cell viability was measured relative to 0 h. Error bars are based on three independent experiments (mean ± SD). (TIFF) [file pone.0225302.s005.tiff]

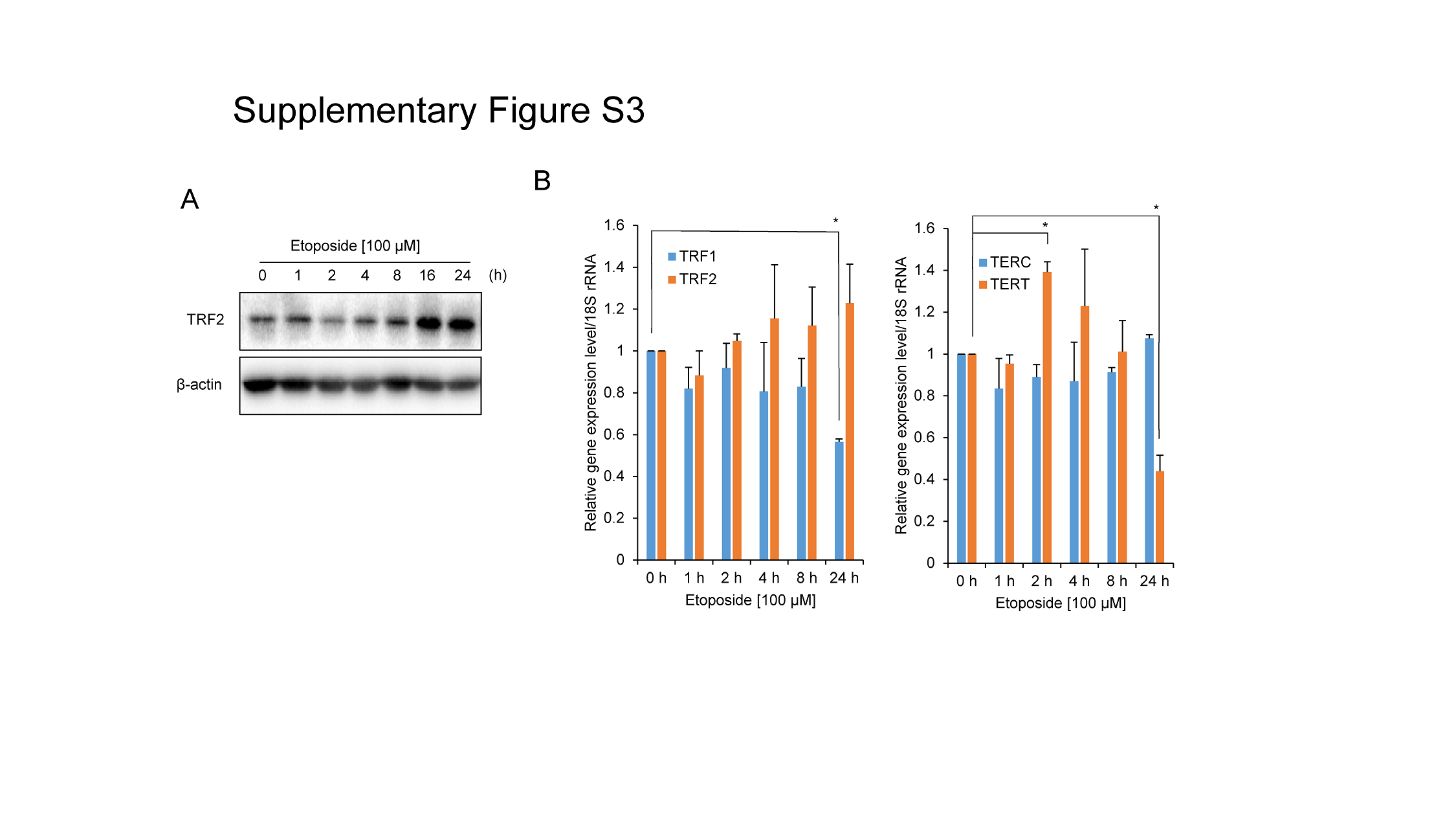

Supplement: S3 Fig — A. Immunoblot for TRF2 in etoposide-treated HeLa cells. Whole-cell lysates prepared from HeLa cells treated with etoposide at 100 μM for the indicated times were subjected to SDS–PAGE followed by immunoblotting. The β-actin was used as a loading control. B. RT–qPCR for telomere-related genes in etoposide-treated HeLa cells. RT–qPCR was performed using HeLa cells treated with etoposide at 100 μM for the indicated times. The gene levels were normalized to 18S rRNA and expressed as fold changes relative to 0 h. Error bars are based on three independent experiments (mean ± SD). Mann-Whitney U-test was used to compare RNA levels between each h and 0 h; *P < 0.05. (TIFF) [file pone.0225302.s006.tiff]

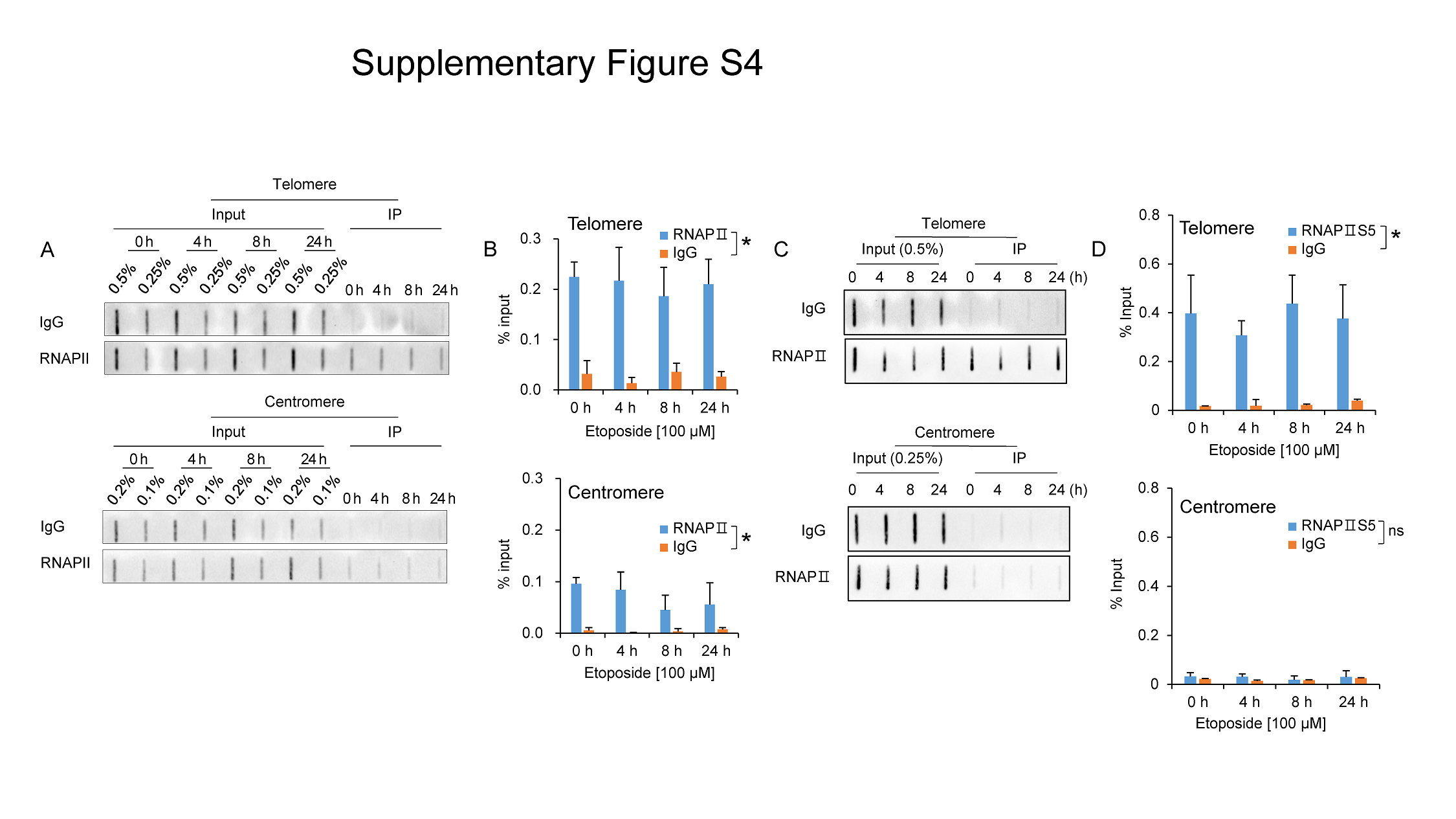

Supplement: S4 Fig — ChIP–slot assays of telomeric and centromeric DNA with an anti-RNAPII antibody and IgG as a control were performed in HeLa cells incubated with etoposide at 100 μM for the indicated times. DNA precipitates were slot-blotted and hybridized with telomeric and centromeric probes. A. ChIP–slot assay using an anti-RNAPII (pS2) antibody. B. Quantification of ChIP–slot assays represented in A (mean ± SD; n = 3). C. ChIP–slot assay using an anti-RNAPII (pS5) antibody. D. Quantification of ChIP–slot assays represented in C (mean ± SD; n = 3). Student’s t-tests were used to compare differences in DNA levels between RNAPII-ChIP and IgG-ChIP; *P < 0.05 and ns indicates P ≥ 0.05. (TIFF) [file pone.0225302.s007.tiff]

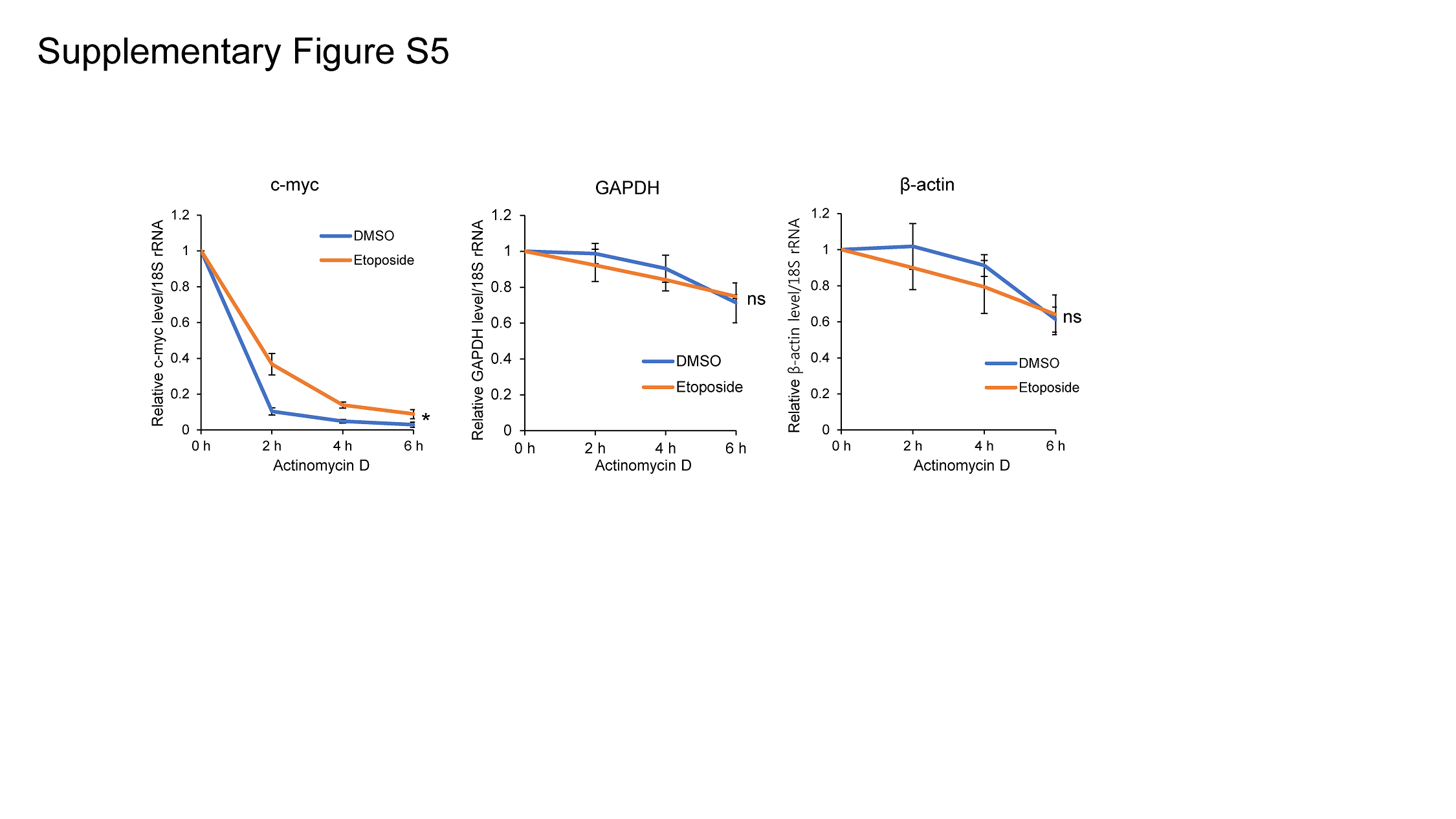

Supplement: S5 Fig — HeLa cells incubated with DMSO (vehicle alone) or etoposide at 100 μM for 24 h were treated with actinomycin D at 5 μg/mL for the indicated times. GAPDH, β-actin, and c-myc were measured by RT–qPCR, normalized against 18S rRNA, and compared with 0 h. Error bars are derived from three independent experiments (mean ± SD). Student’s t-test was used to compare differences in gene levels between etoposide- and DMSO-treated cells; *P < 0.05 and ns indicates P ≥ 0.05. (TIFF) [file pone.0225302.s008.tiff]
